# Sequential Bioprocesses for Biovalorization of Shrimp Pond Sludge by Hydrolytic Enzymes-Producing Bacterial Consortia and Photosynthetic Bacteria

**DOI:** 10.4014/jmb.2501.01042

**Published:** 2025-05-15

**Authors:** Chutema Thongsongkaew, Benjamas Cherisilp, Asma Billateh, Wageeporn Maneechote, Sirasit Srinuanpan

**Affiliations:** 1 Program of Biotechnology, Center of Excellence in Innovative Biotechnology for Sustainable Utilization of Bioresources, Faculty of Agro-Industry, Prince of Songkla University, Hat Yai, Songkhla 90110, Thailand; 2 Center of Excellence of Microbial Diversity and Sustainable Utilization, Faculty of Science, Chiang Mai University, Muang Chiang Mai, Chiang Mai 50200, Thailand

**Keywords:** Aquaculture sludge, amylase, lipase, protease

## Abstract

This study aimed to valorize shrimp pond sludge through sequential bioprocesses using hydrolytic enzyme cocktails produced by bacterial consortia, and photosynthetic bacteria. The production of enzyme cocktails by a co-culture of protease-, amylase-, and lipase-producing bacteria (PAL) was performed in a 5-L stirred tank bioreactor using a low-cost medium. The crude enzyme cocktails were concentrated and used to treat shrimp pond sludge. The addition of enzyme cocktails at 2.0 U/ml based on protease activity led to a reduction of total suspended solids by 40.1% and an increase in soluble chemical oxygen demand (COD) by 3 folds. The solubilized nutrients from shrimp pond sludge in liquid fraction were used as a sole nutrient source to cultivate a newly isolated photosynthetic bacteria (PSB) identified as *Rhodocista pekingensis*. This PSB was able to grow and achieve a high biomass of 1.30 ± 0.28 g/l and produce value-added bioproducts including aminolevulinic acid (11.77 ± 0.55 μM), carotenoids (166.84 ± 0.03 mg/g dry cell weight), and bacteriochlorophylls (771.47 ± 0.17 mg/g dry cell weight). These results highlight the potential use of enzyme cocktails produced by the co-culture of hydrolytic bacteria to facilitate the biovalorization of aquaculture sludge by PSB and may also greatly contribute to biovalorization of other similar aquaculture wastes into valuable bioproducts.

## Introduction

Biovalorization is an effective and increasingly widespread process for value-adding or upcycling byproducts and wastes. Currently, there is an increasing tendency to utilize the wastes from agricultural and food industries as low-cost nutrient sources for the production of microbial enzymes and biochemicals. These wastes are generated in large amounts while having valuable compounds like proteins, carbohydrates, fats, fiber, amino acids, and minerals, making them valuable resources for bioprocesses [1]. Various microorganisms produce enzymes with proteolytic, amylolytic, and lipolytic activities, which play an essential role in the biovalorization of various organic wastes. Microbial enzymes are preferred over enzymes from plants and animals because they are more stable and can me*et al*l the properties desired for biotechnology industries. Microorganisms can also produce enzymes on low-cost fermentation substrates within shorter fermentation cycles. Remarkably, bacterial enzymes are often chosen over other microbial enzymes due to their high productivity, easy availability, and usefulness in biochemical processes [2]. Additionally, the co-cultures of bacteria have also been established to produce microbial enzymes. The advantages of the co-culture would be as follows: i) the co-culture could produce enzyme cocktails with multiple hydrolytic activities using low-cost substrates in one reactor; ii) it is time-efficient; and iii) cost-effective. Moreover, it has also been reported that the co-culture of bacteria can also share metabolic activities, *i.e.*, multiple enzymes that synergistically facilitate the sequential degradation and exchange of metabolites [3].

Most shrimp pond sludge is organic residues which come from the feed left over and the waste excreted by the shrimp. It contains highly nitrogenous compounds like protein, nitrate, nitrile, and ammonia, dissolved organic carbon, and phosphorus. These compounds may cause significant environmental pollution [4]. These wastes negatively affect water quality and disrupt shrimp growth. The traditional way to treat aquaculture sludge is by drying and adding lime. However, the addition of lime may cause problems, leading to incomplete degradation of organic matter. Therefore, environmental problems from shrimp culture must be solved and managed appropriately. Alternatively, biological treatment has been introduced as a more environmentally friendly method. For example, Ramu *et al*. [5] reported the selection of three bacteria strains of *Bacillus* spp. with superior activities of extracellular amylase, protease, and cellulase enzymes. They found that the co-culture of these bacteria did promote the biodegradation of organic matter in shrimp culture water. In our previous report, protease-producing *Exiguobacterium indicum* was isolated and co-cultured with amylase-producing *Bacillus coagulans* and lipase-producing *Bacillus subtilis*. Their co-culture gave higher activities of protease and amylase but with lower activity of lipase compared to the pure culture [6].

Several studies have attempted the enzymatic treatment of sludge. Elsamadony [7] reported that the enzymatic treatment of sludge using enzymes from papaya could increase solubilization efficiency of sludge. Tongco *et al*. [8] also attempted the enzymatic treatment of primary sludge using protease and lipase enzymes and found that the mixed enzymes could improve reduction of volatile suspended solids. In addition to traditional biological treatment, photosynthetic bacteria (PSB) have been used to treat various types of wastewater while producing high-value products like 5-aminolevulinic acid (ALA), a substance with potential applications as a plant growth promoter, herbicide, and pesticide, and bacterial pigments [9]. However, as PSB does not possess hydrolytic activity toward the organic matter in the sludge, to date, there has been no study on the biovalorization of shrimp pond sludge by PSB.

This study aimed to sequentially biovalorize shrimp pond sludge by using hydrolytic enzyme cocktails produced by bacterial consortia to solubilize nutrients in the sludge, and PSB was chosen as model microbes in order to produce a practical biofertilizer containing ALA from solubilized sludge. A co-culture of protease-, amylase-, and lipase-producing bacteria (PAL) was performed in a 5-L stirred tank bioreactor using a low-cost medium. The aeration rate was optimized to maximize enzyme production. Subsequently, the enzyme cocktails were recovered and concentrated. The shrimp pond sludge was then solubilized by the addition of enzyme cocktails and used as a sole nutrient source for cultivation of a newly isolated PSB to produce high-value products including ALA and bacterial pigments.

## Materials and Methods

### Hydrolytic Bacterial Strains

Three hydrolytic bacterial strains, *Exiguobacterium indicum* SSP-PA-08, *Bacillus coagulans*, and *Bacillus subtilis* were used as protease, amylase, and lipase producers, respectively. They were attained from the culture collection at Faculty of Agro-Industry, Prince of Songkla University, Thailand.

### Enzyme Production in a Stirred Tank Bioreactor

The bacteria were pre-cultured in nutrient broth (NB) medium containing 0.1% glucose, 0.3% yeast extract, 1.5% peptone, and 0.6% sodium chloride, at 30 ± 2°C under shaking condition at 150 rpm for 24 h. The low-cost production medium was prepared by using a suspension of shrimp feed (Manee samute farm, Thailand) at 1.2%w/v corresponding to 0.8% total suspended solid (TSS) and adjusted pH to 7.0. The shrimp feed is composed of 42% proteins, 37% carbohydrates, and 6% lipids. The production medium was added to a 5-L stirred tank bioreactor (MDFT500, B.E. Marubishi, Thailand). Subsequently, each bacterium was inoculated to obtain an initial cell concentration of 10^6^ cells/ml. The fermentation was performed under different aeration rates of 1, 2, and 3 air volume per liquid volume per min (vvm) at 150 rpm for 24 h. The samples were collected every 6 h to measure enzyme activity and soluble protein content.

### Recovery of Hydrolytic Enzyme Cocktails

After fermentation in the bioreactor, the cell pellets were separated from the culture broth by centrifugation at 8,000 rpm for 15 min. The cell-free broth was used to precipitate crude enzymes via two methods of ammonium sulfate and acetone precipitation. For ammonium sulfate precipitation, the ammonium sulfate was gradually added to the cell-free broth kept in an ice bath at 4°C up to 80% saturation. After 30 min of keeping at 4°C, the saturated solution was centrifuged at 8,000 rpm for 15 min. The precipitated crude enzymes were dissolved using 0.02 M Tris-HCl buffer, pH 6.8. The obtained enzyme cocktails was then dialyzed using a dialysis bag with 8,000 Da molecular weight cutoff (MWCO) and 0.01 M Tris-HCl buffer, with a volume of 3 L for 3 h. After 3 h, the buffer was changed to 5 L, and dialysis was continued overnight. The enzyme activities and soluble protein content of the enzyme cocktails were measured. For acetone precipitation, acetone was prepared by freezing at -20°C overnight. Then, the supernatant was slowly added with acetone at a ratio of 1:3 and stirred with a stirrer at 4°C for 30 min before centrifugation at 8,000 rpm for 15 min. Protein precipitates were dissolved using 0.02 M Tris-HCl buffer, pH 6.8. The obtained enzyme cocktails were then used to measure enzyme activities and soluble protein content [10].

### Application of Enzyme Cocktails to Solubilize Shrimp Pond Sludge

The sludge that accumulated at the bottom of the shrimp pond (Manee samute farm, Thailand) was collected. The appropriate concentration of enzymes from 2.3 was added to non-sterile shrimp pond sludge containing 6%TSS. Enzyme treatments were performed using various enzyme concentrations: 0, 1.5, and 2.0 U/ml based on protease activity. The experiments were conducted in 125-ml flasks, incubated at 30 ± 2°C and 150 rpm for 48 h. The collected samples were measured for enzyme activities, TSS, soluble protein content, and COD.

### Cultivation of Photosynthetic Bacteria in Enzymatically Treated Sludge

Potential photosynthetic strain was isolated from red-color liquid fertilizer using the spread plate technique. After serial dilution in sterile distilled water, the diluted samples were plated on G5 medium plates (peptone 5 g/l, yeast extract 5 g/l, glutamic acid 4 g/l, malic acid 3.5 g/l, monopotassium phosphate 0.12 g/l, dipotassium phosphate 0.18 g/l and agar 15 g/l). After incubation under anaerobic condition with light intensity of 2,500-3,000 lux and at 30 ± 2°C for 7 days, pink or red colonies (putative PSB candidates) forming distinct morphology were picked and transferred to new G5 medium plates using single colony isolation technique. The red colonies were re-streaked for purification until there was evident uniformity of colony growth on the plates. The identification of photosynthetic bacterial strains was performed based on 16S rRNA sequencing. PSB was cultivated in the liquid fraction of enzymatically treated sludge and synthetic shrimp pond wastewater, which was prepared by using a suspension of shrimp feed (Manee samute farm) at 1.2% w/v corresponding to 0.8% TSS and adjusted pH to 7.0. The heat-deactivated liquid fraction of enzymatically treated shrimp pond sludge was also used as the control. The cultivation was conducted under anaerobic conditions under light intensity of 2,500-3,000 lux and at 30 ± 2°C for 7 days. The samples were taken to measure OD, pH, dry cell weight, ALA, carotenoid content, and bacteriochlorophyll content [11, 12].

### Analytical Methods

**Enzyme activities.** The cell pellets were separated from the culture broth by centrifugation at 8,000 rpm for 15 min. The protease activity in the supernatant was determined following the modified method of Pokhrel *et al*.[13]. Briefly, 130 μl of substrate solution containing 0.65% casein in 0.05 M Tris-HCl buffer, pH 7.5, was added to 25 μl of sample solution. The hydrolysis reaction was conducted at 37°C for 10 min and stopped by adding 130 μl of Trichloroacetic acid (TCA) 0.11 M and incubating at 37°C for 30 min. 250 μl of the supernatant was added to 625 μl of 0.5 M sodium carbonate and mixed thoroughly. Then, the mixture was added with 125 μl of Folin phenol reagent and incubated at 37°C for 30 min. The absorbance at 660 nm of the mixture was measured. The enzyme activity was calculated using tyrosine (Sigma-Aldrich Production GmbH, Switzerland) standard curve. One unit of proteolytic activity was defined as the amount of enzyme that releases 1 μmole tyrosine per min.

The amylase activity was determined by the DNS method [14]. 500 μl of substrate solution containing 1% w/v soluble starch in 0.05 M phosphate buffer, pH 7 was added to 500 μl of the sample. The reaction was performed in water bath at 50°C for 10 min. The mixture was then added to 300 μl of 3,5-dinitrosalicylic acid solution and heated in boiling water for 10 min before being rapidly cooled in ice water. After cooling, 1.6 ml of distilled water was added, and the absorbance was measured at 540 nm. The released glucose was calculated using glucose (Sigma-Aldrich Production GmbH) standard curve. One unit of amylolytic activity was defined as the amount of enzyme required to release 1 μmole of glucose per min.

The lipase activity was determined according to the method reported by Louhasakul and Cheirsilp [15]. 500 μl of substrate solution containing 10% w/v palm oil in iso-octane and 200 μl of 0.050 M phosphate buffer (pH 7.0) were added to 200 μl of the sample. The reaction mixture was vigorously mixed on a high-speed vortex at 10,000 rpm at 30 ± 2°C for 30 min. Then, to stop the reaction, 200 μl of 6 M hydrochloric acid was added, and the upper layer phase was immediately taken. Before measurement, the upper layer phase was suitably diluted with iso-octane and added with 0.4 ml of cupric acetate-pyridine reagent. This mixture was immediately mixed for 15 s and then the absorbance of the mixture was measured at 715 nm. The amount of released palmitic acid was used to calculate lipolytic activity. One unit of lipolytic activity was defined as the amount of enzyme required to release 1 μmole of free fatty acids in the form of palmitic acid per min.

**Soluble protein content, TSS and soluble COD analysis.** After the separation of cell pellets by centrifugation, the soluble protein content in the supernatant was analyzed [16]. The sample solution was properly diluted and added to a test tube that contained Solution A (a mixture of 1% sodium potassium tartrate, 1% CuSO_4_·5H_2_O, and 2% Na_2_CO_3_ in 0.1 M NaOH, in a 1:1:98 ratio) at 1:10 v/v. The mixture was incubated at 30 ± 2°C for 10 min, and Solution B (Folin-Ciocalteau reagent mixed with water in a 1:1 ratio) was added to the sample at a ratio of 1:1 v/v and further incubated at 30 ± 2°C for 30 min. The absorbance was measured at 660 nm and used to calculate soluble protein content as compared with the standard curve.

TSS was measured using the dried filter paper method. First, the filter paper was dried at 103-105°C in an oven for 1 h and weighed after cooling down. The filter paper was placed in a Buckner filter funnel connected to a vacuum pump. A 10 ml sample was poured onto the filter paper, and the vacuum pump was activated until the liquid had been removed. The filter paper with the sample was then dried at 103-105°C in an oven for 1 h. After drying, the filter paper was cooled and weighed to determine the TSS content.

To measure COD, 5 ml of the sample was added to a 20 × 150 mm COD digestion tube that had been pre-washed with 20% H_2_SO_4_. Then, 3 ml of 0.1 N potassium dichromate digestion reagent was added slowly, followed by gentle mixing. Afterward, 7 ml of sulfuric acid reagent mixed with AgSO_4_ was carefully added along the sides of the tube, allowing it to flow to the bottom. The tubes were transferred to a pre-heated COD block digester, and the sample was digested at 150°C for 2 h. Blanks are prepared by substituting distilled water for the sample and processed in the same manner. After digestion, ferroin was added as an indicator in 1–2 drops. The solution was then titrated using 0.1 M ferrous ammonium sulfate (FAS) until the blue-green color changed to reddish-brown color [17].

**Cell growth and metabolites of photosynthetic bacteria.** The cell growth was monitored by measuring optical density at 660 nm (OD_660_) using a spectrophotometer (U-2000, Technical Co., USA). Carotenoids and bacteriochlorophyll analysis were performed followed the method of Hirayama [18]. Briefly, the cell pellets were recovered by centrifugation at 8,000 rpm for 15 min and 1 ml of a methanol-acetone solution (2:3 v/v) was added. The pigments were extracted using ultrasonic treatment for 30 min. The extractant was recovered through centrifugation at 10,000 rpm for 15 min and measured at wavelengths of 480 nm and 770 nm. The amounts of carotenoids and bacteriochlorophyll were calculated. The concentration of ALA was determined using a colorimetric method as outlined by Lin *et al*. [11]. For ALA analysis, the supernatant was added to a test tube together with 1 M acetate buffer (pH 4.6) and acetylacetone at a ratio of 1:2:0.05 v/v. The reaction mixture was heated with boiling water for 15 min. After cooling down, an equal amount of Ehrlich's reagent was added. After mixing well, the solution was left to react at 30 ± 2°C for 15 min, and the resulting color was measured using a spectrophotometer at 553 nm wave length, with the ALA concentration calculated compared with the ALA (Sigma-Aldrich Production GmbH) standard curve.

### Statistical Analysis

All the experimental sets were conducted in triplicate. Duncan’s multiple range test (DMRT) and one-way analysis of variance (ANOVA) were used to evaluate significant differences between treatments (*p* ≤ 0.05).

## Results and Discussion

### Enzyme Production in a Stirred Tank Bioreactor

**Fig. 1:**
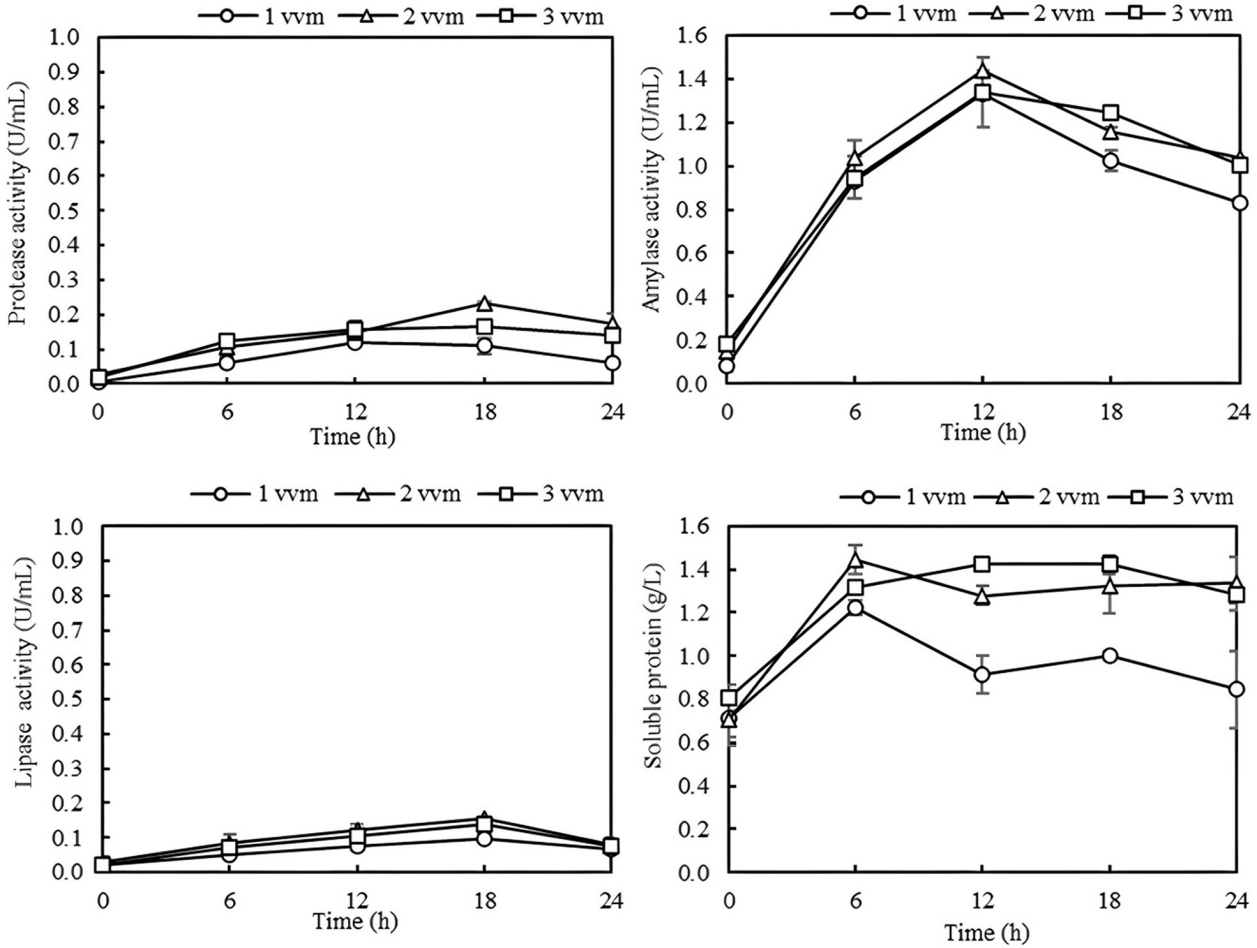
Effect of aeration rate on enzyme production and soluble protein in stirred tank bioreactor.

The production of microbial extracellular enzymes in bioreactors is influenced by various physical and environmental parameters, as well as the chemical composition of the production medium [19, 20]. In this study, the enzyme production was conducted in a 5-L stirred tank bioreactor using co-culture of *E. indicum* SSP-PA-08, *B. coagulans*, and *B. subtilis* as protease, amylase, and lipase producers, respectively. It should be noted that each enzyme activity was measured at their optimal temperatures to estimate the amount of each enzyme present in the culture broth. The actual impact of each enzyme on sludge solubilization may differ from the activity values. Notably, aeration is a crucial parameter that significantly affects enzyme production. Aeration not only provides oxygen for the aerobic fermentation process but also contributes to the homogeneity of the culture broth [21]. The effect of the aeration rate was evaluated by varying aeration rates at 1, 2, and 3 vvm. As shown in Fig. 1, an aeration rate of 2 vvm yielded the highest activities of enzyme cocktails. The slight decrease in amylase activity might be due to protease-mediated degradation. It can be observed that the aeration rate beyond 2 vvm did not further increase enzyme production. It has been reported that the high aeration rate may induce the generation of reactive oxygen species (ROS) in excess, which then inhibits cell growth and restricts enzyme production [22]. Moreover, protein carbonylation caused by ROS may lead to chemical modifications in the produced enzymes, resulting in reduced activity [23]. Another phenomenon known as 'impeller flooding,' where lower agitation speeds combined with higher aeration flows cause the air stream in the bioreactor to rise along the stirrer shaft, results in poor mixing, reduced oxygen transfer rates, and decreased air dispersion [24].

There are very few reports on scaling up hydrolytic enzyme production in bioreactors. Our observations align with the study of Ullah *et al*. [25], who studied the alkaline protease production by locally isolated *Bacillus cereus* AUST 7 in a bioreactor using tannery waste as substrate. They found that the maximum alkaline protease production was achieved at an aeration rate of 2 vvm, stirring speed of 500 rpm, and 24 h incubation period. Suyotha *et al*. [26] also reported that the chitosanase production by *Lentzea* sp. OUR-I1 in a 3-L bioreactor increased sharply when the aeration rate was increased up to 2 vvm and dramatically decreased when the aeration rate exceeded 2 vvm. For amylase production, Deljou *et al*. [24] performed the scaling up of thermostable amylase production in lab-scale fermentor using rice husk as an elicitor by *Bacillus licheniformis*-AZ2. The maximum amylase production was achieved when using the aeration rate of 1 vvm. The aeration rate over 1 vvm caused a significant reduction in both cell growth and enzyme production. While Bakri *et al*. [27] found that amylase production using *Bacillus subtilis* SY134D achieved the maximum enzyme production of 127 IU/ml when using an aeration rate of 0.25 vvm and an agitation speed of 300 rpm in a 3-L bioreactor. Mostafa *et al*. [20] observed that amylase production by *Aspergillus flavus* from water hyacinth was maximal at 0.5 vvm aeration rate and 200 rpm agitation speed. It seemed that the optimal aeration rates for bacterial enzyme production were in the range of 0.25-2 vvm. In this study, the optimal aeration rate for three enzymes was found to be 2 vvm.

### Recovery and Concentration of Enzyme Cocktails

To recover and concentrate the enzyme cocktails, two methods were employed: precipitation using ammonium sulfate and precipitation using acetone (Table 1). The first method involved precipitating 200 ml of crude enzyme extract with ammonium sulfate within a saturation range of 10-80%, followed by desalination through dialysis. This method increased protease activity up to 17.11 U/ml, with a yield of 33.95%. In the case of amylase and lipase, it was also found that the amylase and lipase activities were also increased up to 9.65 U/ml and 0.12 U/ml, respectively. As protease activity was dominant in the enzyme cocktail, it was then used as a representative indicator for expressing the efficiency of the precipitation method. Based on specific protease activity before and after ammonium precipitation (13.26 U/mg and 14.97 U/mg), the purity of the protease enzyme was increased by 1.13 folds. When ammonium sulfate displaces water from the solvation layer of the protein, its ability to interact with water decreases, leading to increased protein-protein interactions and subsequent precipitation [28]. Several studies also showed that ammonium sulfate could precipitate enzymes and achieve a high recovery yield of 25-47% [29, 30]. However, it is also important to emphasize that each protease has unique properties, different sources lead to distinct ammonium sulfate precipitation profiles [31].

Our result revealed that the protein precipitation using acetone increased protease activity only up to 2.81 U/ml but with an increased specific protease activity of 1.37 folds. However, this method resulted in a very low recovery yield of 11.15%, making precipitation with acetone unsuitable for large-scale protein recovery. It was also found that the acetone precipitation could not increase amylase and lipase activities. Therefore, ammonium sulfate precipitation was chosen, as it led to a higher increase in both enzyme activity and enzyme concentration. Additionally, ammonium sulfate has several advantages over other electrolyte compounds, including high solubility, minimal impact on enzyme precipitation, stability under varying pH conditions, and low cost [32]. Similar results have been reported by de Melo Oliveira *et al*. [33], who described the separation and partial purification of collagenolytic proteases obtained from peacock bass using precipitation and partitioning methods. In their study, the 30–60% ammonium sulfate fraction was shown to be the most efficient method for separation compared to acetone and ethanol. Unlike previous studies, Murthy *et al*. [34] used fractional precipitation with acetone, ethanol, and ammonium sulfate to recover visceral proteases from tilapia (*Oreochromis mossambicus*), catla (*Catla catla*), and mackerel tuna (*Euthynnus affinis*). All fish enzymes showed improved recovery rates with acetone.

### Application of Hydrolytic Enzyme Cocktails for the Treatment of Non-Sterile Shrimp Pond Sludge

**Fig. 2:**
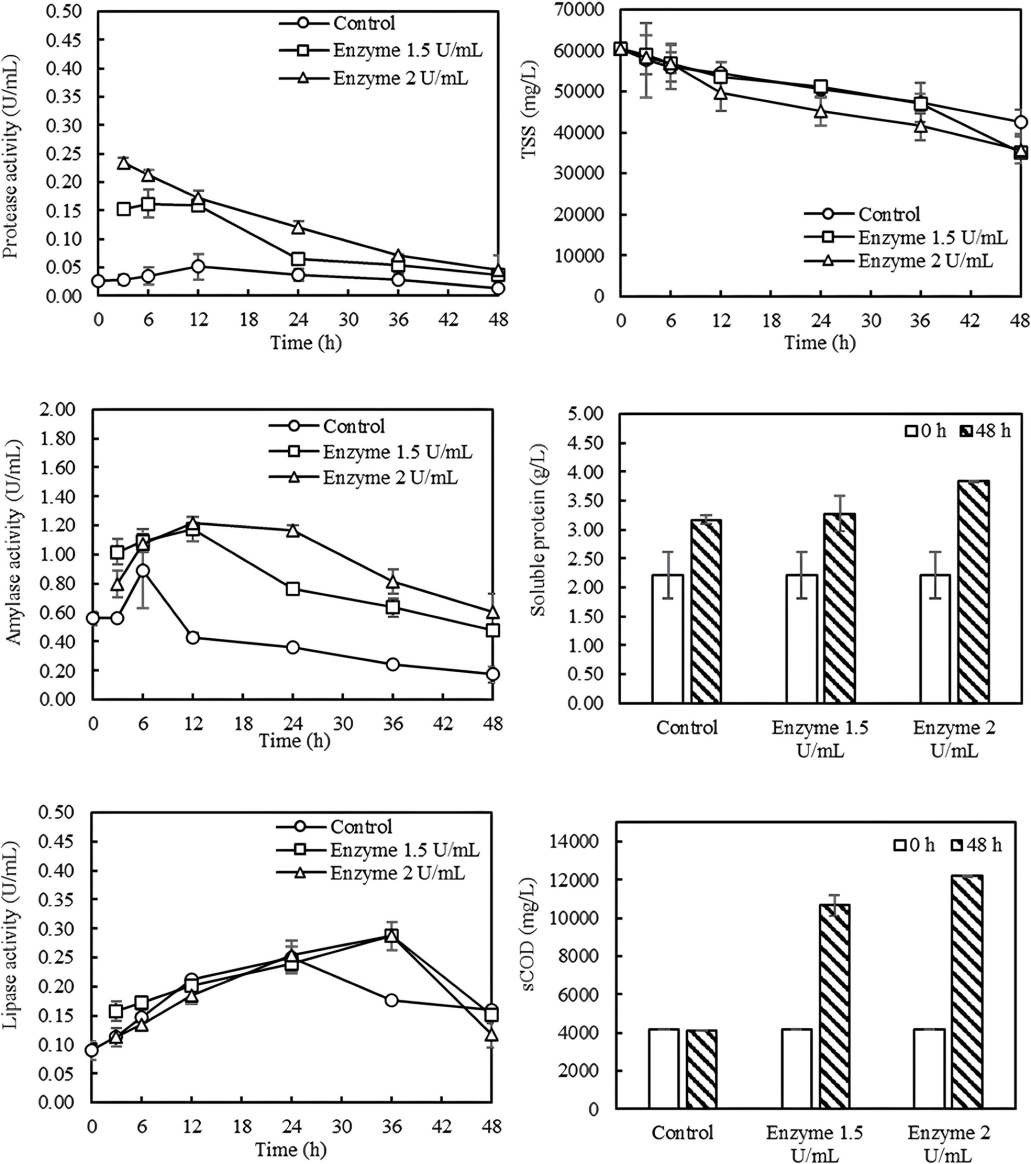
Enzymatic treatment of shrimp pond sludge wastewater. The partially purified hydrolytic enzymes were added at 0, 1.5, 2.0 U/ml protease-based activity and incubated at 30 ± 2°C for 48 h. TSS: total suspended solids, sCOD: soluble COD.

This study investigated the use of hydrolytic enzyme cocktails to treat non-sterile shrimp pond sludge containing 6% TSS. The hydrolytic enzyme cocktails were added at 0, 1.5, and 2.0 U/ml based on protease activity. Fig. 2 shows the enzymatic treatment of shrimp pond sludge wastewater. It should be noted that the sludge without enzyme addition also showed hydrolytic activity indicating the presence of hydrolytic indigenous bacteria in the sludge. During the prolonged incubation time, the activity of protease and amylase decreased, but that of lipase significantly increased. The enzyme treatment reduced TSS by 40.0 ± 0.7%, which was higher than the control without enzyme addition (30.0 ± 2.9%). With the activity of hydrolytic enzyme cocktails, the soluble COD increased from 4,189 mg/l to 12,186 mg/l. It was observed that as the enzyme concentration increased, the soluble COD also increased, whereas the control had unchanged soluble COD. The soluble protein also increased with increasing enzyme loading indicating the effective solubilization of sludge by the enzymatic treatment. As the control also showed an increase in the soluble protein, the reduction in TSS in the control may be primarily due to the solubilization of proteins rather than other organic matter.

Elsamadony [7] reported that the use of enzymes derived from papaya to digest sludge resulted in increased solubilization efficiency of total COD. They also reported that using a mixture of papain, protease, and lipase at a ratio of 3:1:2, with an optimal enzyme concentration of 8% did dissolve more sludge. In addition, Tongco *et al*. [8] studied the improvement of primary sludge digestion using protease and lipase enzymes by mixing the sludge and enzymes and incubating at 40°C and pH 7.0 for 72 h. They found that the enzymatic treatment of primary sludge using mixed protease to lipase ratio of 1:3 resulted in a reduction of volatile suspended solids by 33.3%. Additionally, this treatment increased the subsequent production of biogas by 84.1%. The sludge treated with these enzymes was effectively used in the anaerobic digestion process, demonstrating that enzyme treatment can reduce suspended solids and increase soluble COD value.

### Cultivation of Photosynthetic Bacteria in Enzymatically Treated Sludge

**Fig. 3:**
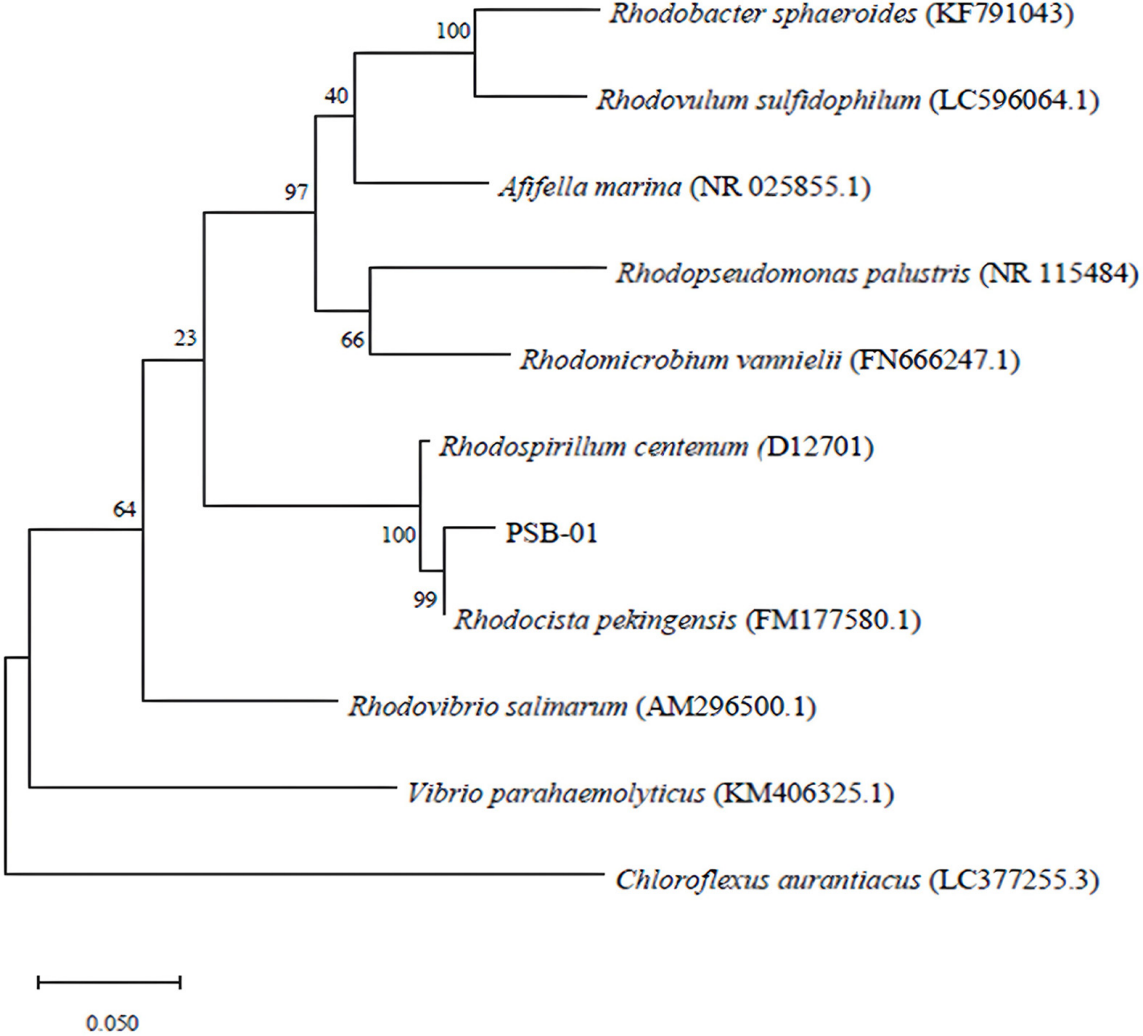
The phylogenetic tree shows the relationship among PSB strains and their phylogenetically closest strains. The GenBank accession numbers of the type strains and the studied strain are shown following species names.

The applications of PSB for the biological treatment of wastewater coupled with the production of value-added products have been widely studied. PSB have the ability to use light as an energy source and use a variety of compounds like carbon, sulfide, and ammonium [9]. However, PSB cannot be used to valorize sludge directly as it does not possess sufficient amount of hydrolytic activities toward sludge. This study aimed to apply PSB for valorizing shrimp pond sludge after enzymatic treatment into valuable products. The newly isolated PSB was identified using 16S rDNA sequencing and homology analysis. The obtained sequence (1,043 bp) was submitted to DDBJ under accession number LC645458. Based on the 16S rRNA gene sequence similarity, the strain was assigned to the genus *Rhodocista*. Blast analysis (http://ddbj.nig.ac.jp/blast/) revealed that the isolate PSB-01 shows the highest sequence similarity (99%) to *Rhodocista pekingensis*. The 16S rRNA gene sequences of strain PSB-01 and closely related species retrieved from DDBJ were aligned using the Clustal W program, and the maximum likelihood method (MEGA version 6.0) was used to reconstruct a phylogenetic tree (Fig. 3). The results indicate that the purple photosynthetic bacterium isolated PSB-01, which does not accumulate sulfur, is *R. pekingensis*. This strain was then employed to be cultivated in solubilized shrimp pond sludge with the aim of producing value-added products.

**Fig. 4:**
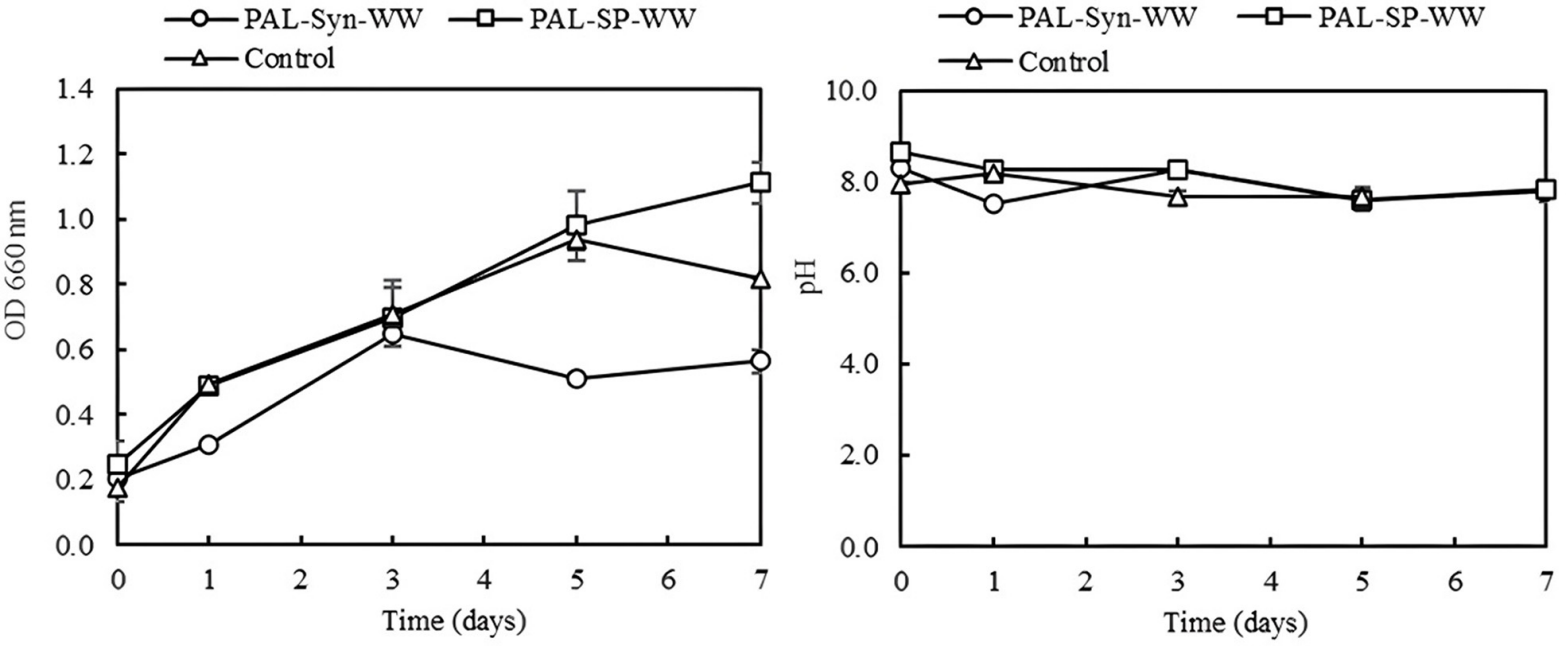
Biomass in terms of optical density (OD at 660 nm) (A) and pH (B) of *R. pekingensis* cultivation in synthetic wastewater and shrimp pond wastewater under 2,500-3,000 light intensity and anaerobic condition at 30 ± 2°C for 7 days. PAL-Syn-WW: Liquid fraction of enzymatically treated synthetic wastewater; PAL-SPWW: Liquid fraction of enzymatically treated shrimp pond sludge; Control: Deactivated liquid fraction of enzymatically treated shrimp pond sludge.

After enzymatic solubilization of shrimp pond sludge, only liquid fraction after centrifugation was used for cultivation of PSB. Three media for PSB were prepared. The first medium was the liquid fraction of the synthetic wastewater treated with hydrolytic enzyme cocktails (PAL-Syn-WW). The second medium was the liquid fraction of the shrimp pond sludge treated with hydrolytic enzyme cocktails (PAL-SP-WW). The third medium was the deactivated liquid fraction of treated shrimp pond sludge (Control). PSB was inoculated at 10% v/v and incubated under anaerobic conditions with 2,500-3,000 lux of light intensity at 30 ± 2°C for 7 days. The results are shown in Fig. 4. It was found that PSB grew better in the liquid fraction from the enzymatically treated shrimp pond sludge (PAL-SP-WW) than that from the enzymatically treated synthetic wastewater (PAL-Syn-WW), possibly due to the more available nutrients in the shrimp pond sludge. There was no significant difference in cell growth between the non-deactivated (PAL-SP-WW) and deactivated (Control) liquid fractions of enzymatically treated shrimp pond sludge during the 5-day cultivation period. However, after 5 days, greater growth was observed with the non-deactivated liquid fraction, suggesting that the enzyme cocktails might still be active and releasing additional nutrients for PSB. Additionally, the pH values remained relatively stable throughout the experiment. The value-added products are detailed in Table 2. *R. pekingensis* could grow well when using the liquid fraction from the enzymatically treated shrimp pond sludge (PAL-SP-WW) and gave the highest biomass of 1.30 ± 0.28 g/l. *R. pekingensis* also produced 11.77 ± 0.55 μM of 5-aminolevulinic acid (ALA). The contents of carotenoids and bacteriochlorophylls were also as high as 166.84 ± 0.03 mg/g-dried cell weight and 771.47 ± 0.17 mg/g-dried cell weight, respectively. It should be noted that light intensity is closely associated with the production of value-added substances, particularly pigments [35]. However, neither culture in synthetic wastewater nor shrimp pond wastewater produced hydrogen (data not shown), likely due to the absence of volatile fatty acids in the media, which are essential for hydrogen production by PSB. These results have shown that PSB has the potential to produce high value-added products using enzymatically treated shrimp pond sludge as a low-cost substrate.

According to Meng *et al*. [12], *Rhodopseudomonas* grew more than 2.6 times when using treated wastewater from breweries with the addition of 0.4 g/l yeast extract. Lu *et al*. [36] reported that brewery wastewater supplemented with nitrogen could replace traditional culture medium while maintaining comparable biomass output. The value-added substances produced in PSB cells grown in brewery wastewater in outdoor pilot-scale photobioreactors included protein, carotenoid, bacteriochlorophyll, and Coenzyme Q10, with concentrations of 1014.4, 4.9, 23.0, and 67.3 mg/l, respectively. Additionally, Patthawaro *et al*. [37] investigated the use of agro-industrial waste for the production of the value-added product carotenoid by the photosynthetic bacterium *Rhodopseudomonas faecalis*. The carotenoid composition of this bacterium grown in soybean meals included lycopene, 1,2-dihydrolycopene, cis-1,2-dihydrolycopene, and 1,2-dihydro-3,4-dedihydrolycopene.

## Conclusion

In this study, the hydrolytic enzyme cocktails were produced by the co-culture of proteolytic-, amylolytic-, and lipolytic-bacteria in the bioreactor with the optimal aeration rate of 2 vvm. The enzyme cocktails were recovered and concentrated using ammonium sulfate precipitation. The concentrated enzyme cocktails were applied to treat shrimp pond sludge. With the enzymatic treatment, the TSS in the shrimp pond sludge wastewater was reduced by 40.0 ± 0.7% with the increase in the soluble protein and soluble COD. Additionally, the newly isolated *R. pekingensis* PSB-01 was used to valorize the treated shrimp pond sludge and achieved biomass of 1.30 ± 0.28 g/l and significant productions of ALA and bacterial pigments. Besides PSB, other microbes might also be cultivated in the solubilized sludge to produce other value-added chemicals. The study has demonstrated a cost-effective way to produce hydrolytic enzyme cocktails and the sequential biovalorization of shrimp pond sludge into high value products.

## Figures and Tables

**Table 1:** Purification procedures for enzyme cocktails.

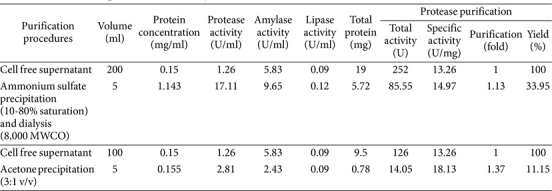

**Table 2:** Value-added products from *R. pekingensis* cultivation in liquid fraction of enzymatically treated synthetic wastewater (PAL-Syn-WW) and that of enzymatically treated (PAL-SP-WW) shrimp pond sludge.

Media	Dry cell weight (g/l)	Pigments (mg/g cell weight)		ALA (μM)
		Carotenoids	Bacteriochlorophylls	
PAL-Syn-WW	0.35 ± 0.0	0.92 ± 0.17	6.20 ± 1.40	20.00 ± 0.55
PAL-SP-WW	1.30 ± 0.28	166.84 ± 0.03	771.47 ± 0.17	11.77 ± 0.55
